# Evaluation of Nd_0.8−*x*_Sr_0.2_Ca_*x*_CoO_3−*δ*_(*x* = 0, 0.05, 0.1, 0.15, 0.2) as a cathode material for intermediate-temperature solid oxide fuel cells†

**DOI:** 10.1039/d2ra02546a

**Published:** 2022-06-13

**Authors:** Xu Du, Songbo Li, Shengli An, Liangmei Xue, Yang Ni

**Affiliations:** School of Chemistry and Chemical Engineering, Inner Mongolia University of Science and Technology Baotou 014000 China songboli2021@hotmail.com; School of Material and Metallurgical Engineering, Inner Mongolia University of Science and Technology Baotou 014010 China

## Abstract

A series of Nd_0.8−*x*_Sr_0.2_Ca_*x*_CoO_3−*δ*_(*x* = 0, 0.05, 0.1, 0.15, 0.2) cathode materials was synthesized by sol–gel method. The effect of Ca doping amount on the structure was examined by scanning electron microscopy (SEM), X-ray diffraction (XRD), thermal expansion, and X-ray photoelectron spectroscopy (XPS). Electrochemical properties were evaluated for possible application in solid oxide fuel cell (SOFC) cathodes. Results showed that second phase NdCaCoO_4+*δ*_ is generated when the Ca doping amount is higher than 0.1. The increase in Ca limits the electronic compensation capacity of the material, resulting in a decrease in thermal expansion coefficient (TEC). With the increase of Ca content, the conductivity increases at first and then decreases, and the highest value of 443 S cm^−1^ is at *x* = 0.1 and *T* = 800 °C. Nd_0.7_Sr_0.2_Ca_0.1_CoO_3−*δ*_ exhibits the lowest area specific resistance of 0.0976 Ω cm^2^ at 800 °C. The maximum power density of Nd_0.7_Sr_0.2_Ca_0.1_CoO_3−*δ*_ at 800 °C is 409.31 mW cm^−2^. The Ca-doped material maintains good electrochemical properties under the coefficient of thermal expansion (CTE) reduction and thus can be used as an intermediate-temperature SOFC (IT-SOFC) cathode.

## Abstract 2

The increase in Ca for Nd_0.8−*x*_Sr_0.2_Ca_*x*_CoO_3−*δ*_ limits the electronic compensation capacity, resulting in a decrease in CTE. The Ca-doped material maintains good electrochemical properties under CTE reduction and thus can be used as an IT-SOFC cathode.

## Graphical abstract

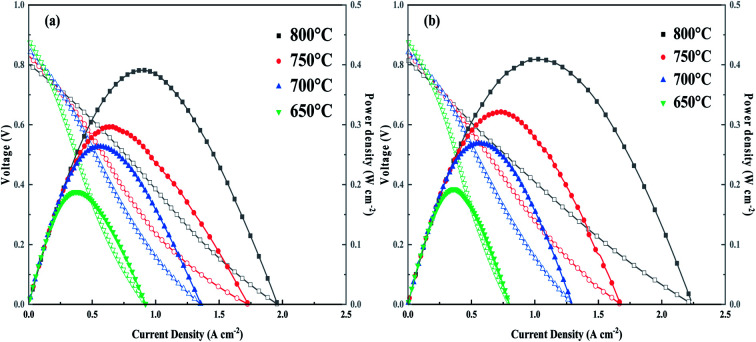

## 1. Introduction

The solid oxide fuel cell (SOFC) is an all-solid-state device for efficient energy conversion at high operating temperature. Despite its extremely impressive energy conversion efficiency, its high operating temperature (800–1000 °C) limits the applications for this technology and thus hinders its further development.^1^ Reducing the operating temperature of SOFCs has become a major research focus for their broad application in this field. The overall performance of SOFCs is mainly influenced by the oxygen reduction reaction (ORR) cathodic activity, which in turn is directly related to temperature.^2^ Therefore, the choice of cathode material influences the overall SOFC performance. In recent years, Perovskite oxide (ABO_3_) with mixed ion–electron conductors (MIEC) has been extensively studied and applied in intermediate-temperature solid oxide fuel cells (IT-SOFCs).^3^ The ABO_3_ structure has a high concentration of oxygen vacancies that maintain the coexistence of many metal ions with different oxidation states, resulting in good ORR catalytic activity.^4^ These characteristics render its potential use in SOFC and solid oxide electrolyzer cells.

Among available materials, LaCoO_3−*δ*_ has attracted attention due to its impressive ORR activity and good proton–ion conductivity.^5^ However, Co-based materials have a high coefficient of thermal expansion (CTE), which results in high thermal stress at high temperature and limits their further development.^6^ High thermal stress can loosen the bond between cathode and electrolyte, leading to cathode detachment and cell failure. As a solution, La has been replaced with elements such as Pr, Nd, Sm, and Gd in LaCoO_3−*δ*_ structure.^7–9^ However, the conductivity and electrochemical properties of LnCoO_3−*δ*_ (Ln = Pr, Nd, Sm, Gd) decrease with ionic radius, and lanthanide shrinkage reduces lattice symmetry and limits the proton conduction of the material.^10,11^ NdCoO_3−*δ*_ exhibits lower electrical conductivity and thermal expansion properties.^12,13^ Compared with La, Nd has a smaller ionic radius, shorter Nd–O bond, lower electrical conductivity, and better thermal stability.^14,15^ Although the CTE of NdCoO_3−*δ*_ is already lower than that of LaCoO_3−*δ*_, this property must be further reduced to the level in electrolytes.^16^ The most common method of improving thermal expansion is to use Sr instead of Nd to enhance the electrochemical properties. Lee *et al.* reported an increase in conductivity and a decrease in CTE with the increasing Sr content for Nd_1−*x*_Sr_*x*_CoO_3−*δ*_.^17^ Similar to Sr doping, the introduction of Ba and Ca into perovskite oxides has been increasingly investigated.^18,19^ In some layered perovskite doped with Ca, oxygen loss is reduced, and phase stability at high temperatures is improved, resulting in improved electrochemical properties and decreased CTE.^20,21^ Therefore, the role of Ca doping in perovskite structure must be extensively examined.

In this work, Ca was used to replace Nd in Nd_0.8−*x*_Sr_0.2_Ca_*x*_CoO_3−*δ*_ (*x* = 0, 0.05, 0.1, 0.15, 0.2) to investigate the effect of Ca content on the crystal structure. Improvements in thermal expansion and electrochemical performance were investigated in detail to examine the feasibility of this material as an IT-SOFC cathode.

## 2. Experimental

### 2.1 Powder preparation

**Fig. 1:**
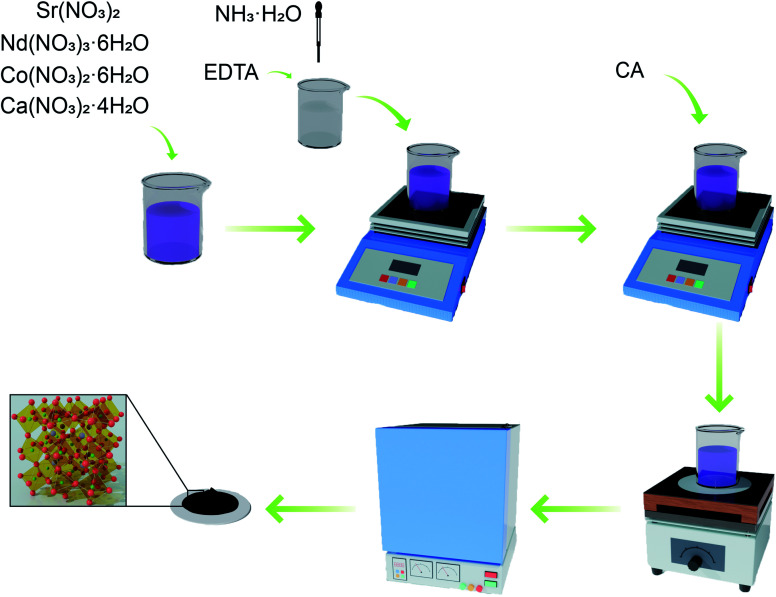
Experimental flow chart of NSC_*x*_C.

Nd_0.8−*x*_Sr_0.2_Ca_*x*_CoO_3−*δ*_(*x* = 0, 0.05, 0.1, 0.15, 0.2) was prepared by EDTA–citric acid (CA) method. Nd(NO_3_)_3_·6H_2_O (Aldrich, 99%), Sr(NO_3_)_2_ (Aldrich, 99.99%), Co(NO_3_)_2_·6H_2_O (Aldrich, 99.99%), and Ca(NO_3_)_2_·4H_2_O (Aldrich, 99%) were dissolved in ammonia and stirred thoroughly. The two complexing agents were weighed in the ratio of metal cation : EDTA : CA = 1 : 1 : 2. EDTA (99.5%) was dissolved in ammonia and added to the above nitrate solution with continuous stirring for 1 h. CA (99.5%) was subsequently added, and the complexation was continued by stirring for 2 h. Solution pH was adjusted to pH ≥ 6 by adding ammonia and heated with stirring to remove excess solvent until a gel was formed. The gel was then heated to spontaneous combustion and calcined in air at 900 °C for 24 h, and the procedure is shown in Fig. 1. Nd_0.8−*x*_Sr_0.2_Ca_*x*_CoO_3−*δ*_ (*x* = 0, 0.05, 0.1, 0.15, 0.2) samples were named NSC, NSC_0.05_C, NSC_0.1_C, NSC_0.15_C, and NSC_0.2_C, respectively.

Gd_0.20_Ce_0.80_O_1.90_ (GDC) powder for the electrolyte was also synthesized by the EDTA–CA method with stoichiometric amounts of metal cation : EDTA : CA = 1 : 1 : 2. The gel was then heated to remove organic products, and the obtained powders were finally annealed at 1250 °C for 5 h. NiO-GDC powder was synthesized through solid-state reaction. NiO (Aldrich, 99%), GDC, and starch with mass ratio of 6 : 4 : 1 were mixed for 15 h in anhydrous alcohol in a zirconia grinding medium. Similar method was used to prepare NSC_*x*_C + GDC hybrid powder by ball milling an equal mass mixture of NSC_*x*_C/GDC and calcining at 900 °C for 5 h. This power was used to investigate the chemical compatibility of NSC_*x*_C and GDC electrolytes.

### 2.2 Cell preparation

Electrolyte-supported symmetric cells were prepared for the electrochemical performance tests. Impedance test was conducted using an electrolyte-supported symmetric cell. The GDC powder was pressed into sheets (15 mm in diameter, 0.5 mm in thickness) under 200 MPa pressure, and the GDC electrolyte support was obtained after calcination in air at 1450 °C for 10 h.

The cathode part of the symmetric cell was prepared by screen printing. NSC_*x*_C powder was mixed with pine oil alcohol (Aldrich, 95%) and ethyl cellulose (Aldrich, 45–55 mPa s) in a mass ratio of 2 : 2 : 1 to obtain NSC_*x*_C cathode slurry, which was then printed onto both sides of the electrolyte symmetry and calcined in air at 900 °C for 5 h to obtain a symmetrical cell with the NSC_*x*_C|GDC|NSC_*x*_C structure.

The electrochemical performance of this cell was tested by using an anode-supported single cell. In brief, 0.15 g of GDC was laid flat on top of 1.5 g of NiO-GDC and pressed under 200 MPa in a *φ* = 15 mm diameter mold to obtain the NiO-GDC|GDC anode supported half-cell. The cathode side was prepared in the same way as the symmetric cell to fabricate a single cell with NiO-GDC|GDC|NSC_*x*_C structure.

### 2.3 Characterization

The XRD (Malvern Panalytical, Empyrean, Cu-Kα radiation) patterns of samples were used to confirm their crystalline structure. The morphologies of the materials and cells were tested by SEM (TESCAN, GAIA3). The CTE of the samples was determined by an L75 HS 1600 thermal expansion meter (NETZSCH). The composition and valence of each element on the surface was studied by XPS using ESCALAB 250Xi instrument (Thermo Fisher Scientific).

### 2.4 Electrochemical performance test

The electrochemical performance of NSC_*x*_C samples was tested in PGSTAT302N electrochemical workstation (Metrohm). Electrical conductivity was simultaneously measured for all samples by a four-terminal DC arrangement in the range of 200–800 °C with Ag paste as the collector. Electrochemical impedance (EIS) tests for NSC_*x*_C|GDC|NSC_*x*_C symmetric cells were carried out at a temperature interval of 650–800 °C, a frequency range of 100 kHz–0.1 Hz, and an amplitude of 10 mV in RMS mode. Single cell output performance for NSC_*x*_C|GDC|NiO-GDC single cells was examined at 650 °C-800 °C with humidified H_2_ (3% H_2_O) as the fuel gas and ambient air as the oxidizing agent.

## 3. Results and discussion

**Fig. 2:**
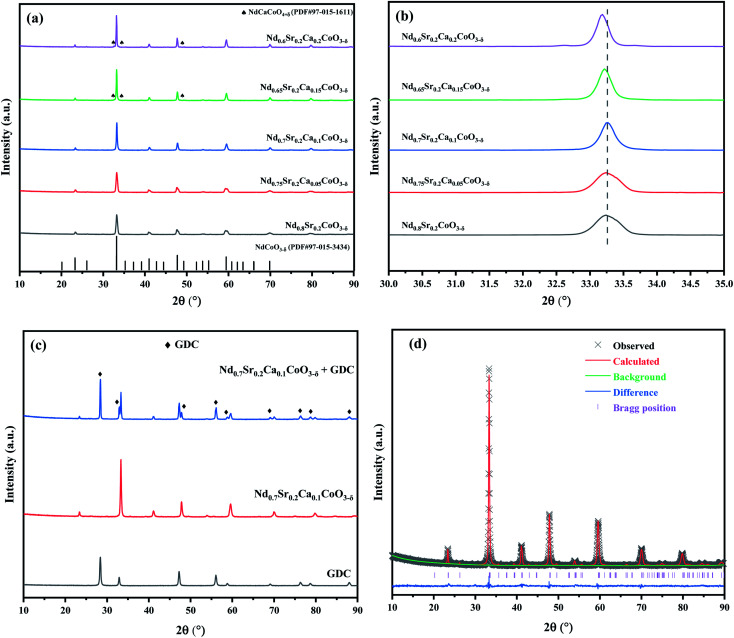
(a) XRD patterns of NSC_*x*_C (*x* = 0, 0.05, 0.1, 0.15, 0.2); (b) magnified XRD patterns of (a) in the range of 30.0 ≤ 2*θ* ≤ 35.0; (c) XRD patterns of GDC, NSC_0.1_C and NSC_0.1_C + GDC; (d) Rietveld refinement of the XRD patterns for NSC_0.1_C.

The XRD patterns of the NSC_*x*_C powders calcined at 900 °C are shown in Fig. 2(a), and the magnification (30.0°–35.0°) is shown in Fig. 2(b). All the curves are generally consistent, but the generation of the second phase NdCaCoO_4+*δ*_ (PDF#97-015-1611) is observed only when the Ca doping amount is above 1.5. When the Ca content increases, the main diffraction peak of the XRD patterns shifts to the left with an increasing degree due to lattice expansion.^22^ The XRD patterns of GDC, NSC_0.1_C, and NSC_0.1_C + GDC are presented in Fig. 2(c). The two materials remain two separate entities after mixing and calcination without the formation of a new component, indicating the good chemical compatibility of NSC_0.1_C and GDC electrolytes. The coordination number of A-site elements in ABO_3_ structure is 12. Comparing the ion radii, it is found that *r*_Ca(ii)_ (1.35 Å) > *r*_Nd(iii)_ (1.27 Å). The effects of increasing Ca content in the crystal structure of NSC_*x*_C are further investigated, and the XRD patterns are analyzed by Rietveld refinement using GSAS + EXPGUI software as shown in Table 1.^23,24^ The Rietveld refined XRD patterns for NSC_0.1_C are shown in Fig. 2(d), and those for the other samples are shown in Fig. S1(a–d).† The results show that the unit cell volume of NSC_*x*_C powders increases gradually with the increase in Ca content.

**Table 1:** Rietveld refinement results for the NSC_*x*_C

Sample	NSC	NSC_0.05_C	NSC_0.1_C	NSC_0.15_C	NSC_0.2_C
Space group	Pbnm	Pbnm	Pbnm	Pbnm	Pbnm
Volume (Å)	215.88	216.74	217.23	217.45	218.19
*a* (Å)	5.35	5.40	5.37	5.37	5.39
*b* (Å)	5.33	5.33	5.33	5.34	5.31
*c* (Å)	7.57	7.54	7.59	7.58	7.62
*χ* ^2^	1.969	2.471	1.714	2.299	2.31
*R*_wp_ (%)	2.86%	4.43%	3.61%	4.15%	4.24%
*R*_p_ (%)	3.32%	3.43%	2.81%	3.21%	3.30%

**Fig. 3:**
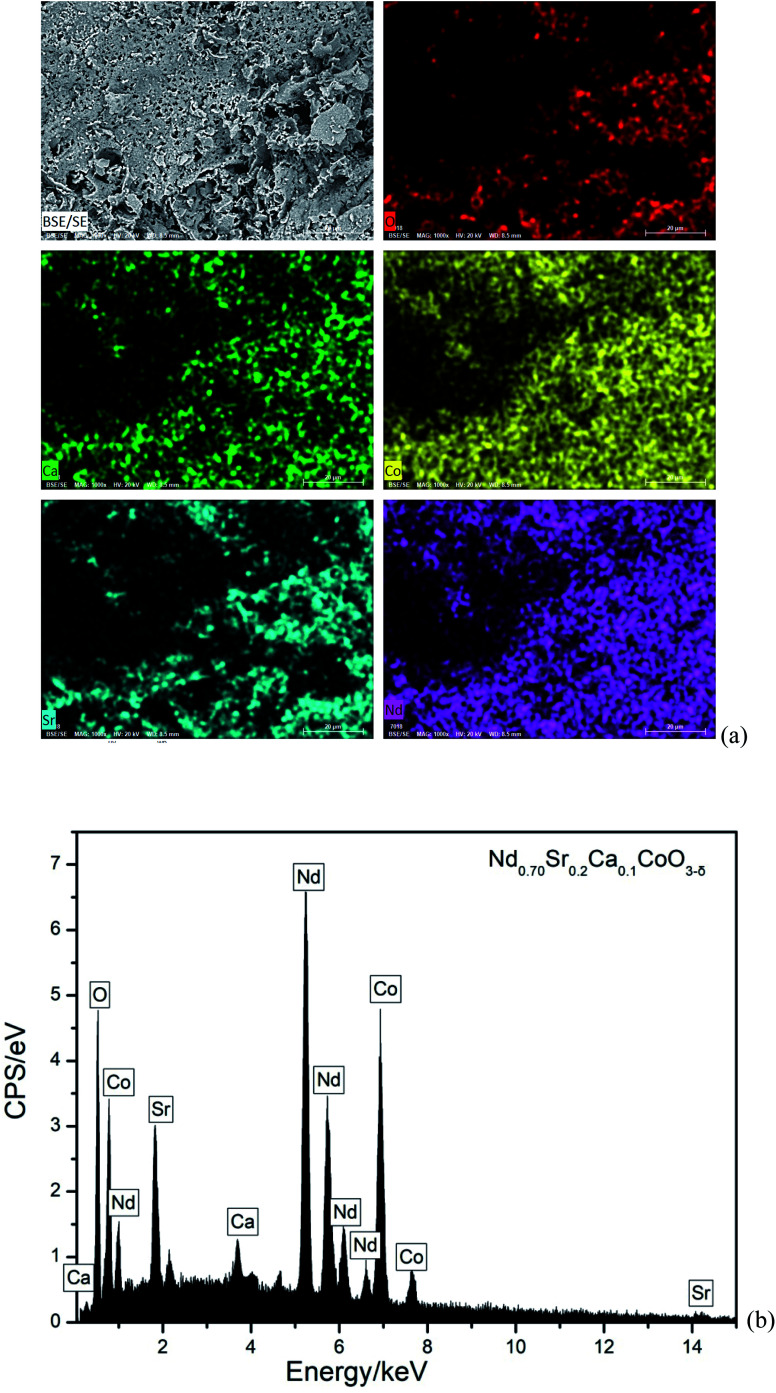
(a) FE-SEM image and elemental mappings of NSC_0.1_C (b) EDS spectrum of NSC_0.1_C.

As a representative, Fig. 3(a) illustrates FE-SEM image and elemental mappings of NSC_0.1_C sample. The morphology of this cathode powder shows a pore structure. Its EDS spectrum is also shown in Fig. 3(b). These reveal that the matrix is made up of Nd, Sr, Ca, Co, and O elements. The elements are homogeneously distributed. Moreover, the atomic content of Nd, Sr, Ca, Co, and O in the Nd_0.8−*x*_Sr_0.2_Ca_*x*_CoO_3−*δ*_ (*x* = 0, 0.05, 0.1, 0.15, 0.2) samples are listed in Table 2. The atomic contents are very close to the theoretical atomic contents, which prove the composite of the prepared Nd_0.8−*x*_Sr_0.2_Ca_*x*_CoO_3−*δ*_ samples.

**Table 2:** Atomic contents of Nd, Sr, Ca, Co, and O in Nd_0.8−*x*_Sr_0.2_Ca_*x*_CoO_3−*δ*_ (*x* = 0, 0.05, 0.1, 0.15, 0.2)

*x*	Atom%					
		Nd	Sr	Ca	Co	O
0	Actual	18.18	4.55	0	22.73	54.55
	Theoretical	18.09	4.42	0	22.78	54.71
0.05	Actual	17.14	4.57	1.14	22.86	54.29
	Theoretical	17.23	4.61	1.08	22.82	54.26
0.1	Actual	16.09	4.6	2.3	22.99	54.02
	Theoretical	16.13	4.55	2.28	23.01	54.03
0.15	Actual	15.03	4.62	3.47	23.12	53.76
	Theoretical	14.92	4.61	3.42	23.09	53.96
0.2	Actual	13.95	4.65	4.65	23.36	53.49
	Theoretical	14.01	4.68	4.61	23.41	53.29

**Fig. 4:**
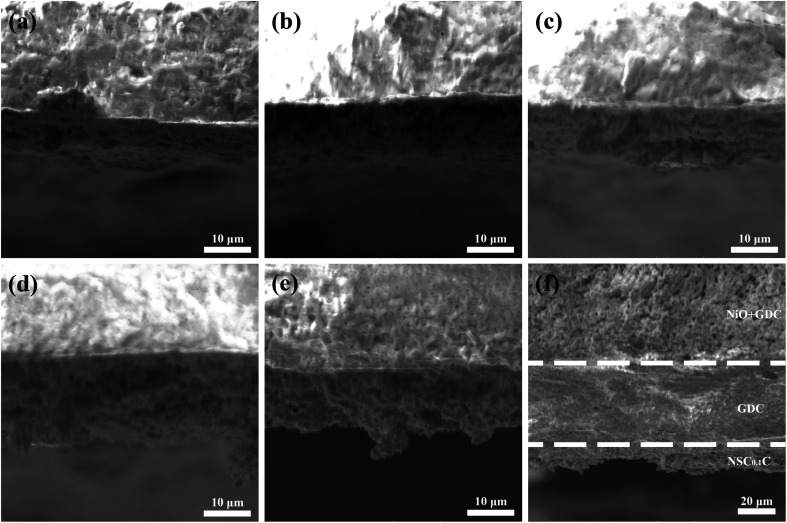
SEM images of the cathode/electrolyte bilayer cross-section (a) NSC|GDC; (b) NSC_0.05_C|GDC; (c) NSC_0.1_C|GDC; (d) NSC_0.15_C|GDC; (e) NSC_0.2_C|GDC; (f) NSC_0.1_C|GDC|NiO-GDC.

The cross-sectional SEM images of the NSC_*x*_C|GDC|NiO-GDC cell with an anode support structure are shown in Fig. 4, and 4(a–e) displays the interface between NSC_*x*_C and GDC connection. The interface between NSC_*x*_C and GDC electrolyte is well defined and in good contact without delamination, indicating the good compatibility between NSC_*x*_C and GDC electrolyte.^25^ A certain amount of closed pores and no open pores are observed in the GDC electrolyte region and can be considered as a dense GDC membrane.^26^ The well-defined interfaces of the layers in NSC_0.1_C|GDC|NiO-GDC in Fig. 4(f) confirm their electrochemical properties. NSC_*x*_C powders do not show uniform grains, indicating that Ca doping has no evident effect on their microstructure.^27^

**Fig. 5:**
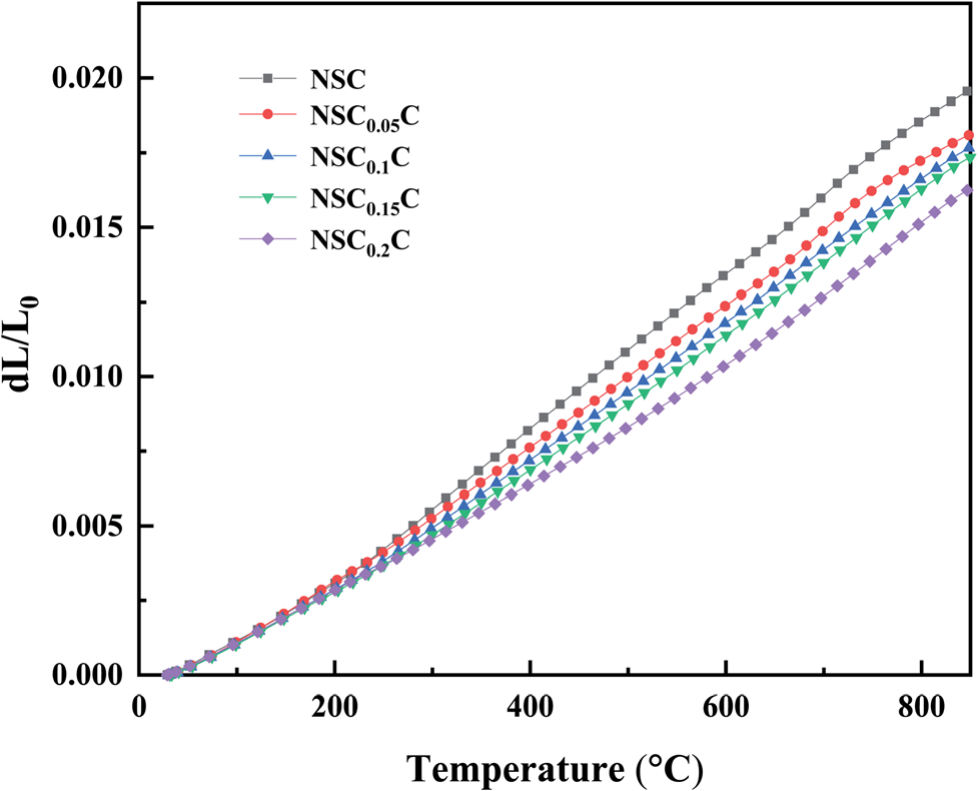
Thermal expansion curves of NSC_*x*_C (*x* = 0.05, 0.1, 0.15, 0.2).

The thermal expansion curves of NSC_*x*_C are shown in Fig. 5. The overall thermal expansion characteristics are suppressed with the increasing Ca doping amount. All samples exhibit non-linear thermal expansion behavior with the increasing temperature. This phenomenon occurs because Co^3+^ at low temperature exists mainly in the form of Co^3+^
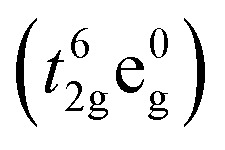
 in the low spin state and gradually shifts to either Co^3+^
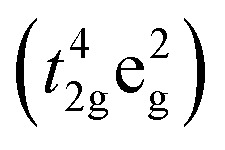
 in the high spin state or Co^3+^
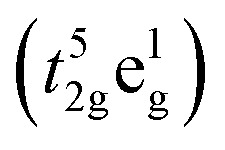
 in the intermediate spin state when the temperature increases; this shift causes the cell to expand.^28^ In addition, the introduction of Sr^2+^ and Ca^2+^ in the NSC reinforces the charge compensation guided by the ionic mechanism, and the elevated temperature further promotes the formation of oxygen vacancies.^4,29,30^ At 200–600 °C, the thermal expansion behavior of the material gradually shifts from non-linear to linear with the increasing Ca content, and the average CTE gradually decreases in all temperature bands from 25.7603 × 10^−6^ K^−1^ for NSC to 19.7623 × 10^−6^ K^−1^ for NSC_0.2_C (35–800 °C). Because Ca stabilizes the spin state of Co, the ionic property of Co–O bond is reduced, the covalent property of Co–O bond is improved, and CTE is reduced. The average CTE for each temperature band is shown in Table S1.† The weakening of CTE by Ca addition is relatively limited within the 600–800 °C range and decreases from 24.2049 × 10^−6^ K^−1^ for NSC to 23.9310 × 10^−6^ K^−1^ for NSC_0.2_C. Compared with its stabilizing effect on Co^3+^ in the intermediate spin state, Ca^2+^ gradually increases its enhancement effect on charge compensation guided by the ionic mechanism at high temperatures. Therefore, Ca doping has a progressively weak effect on the CTE of NSC at the range of 600–800 °C.^27,31^

**Fig. 6:**
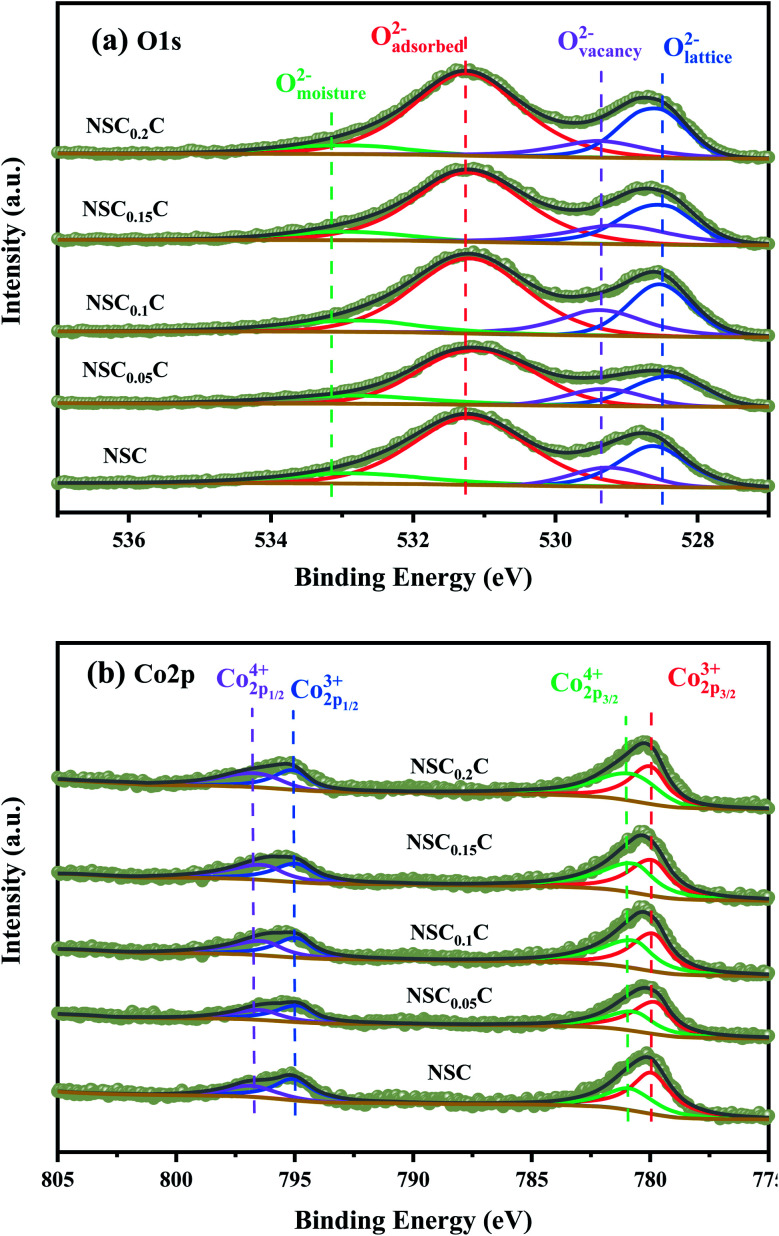
Fitted XPS spectra of NSC_*x*_C(*x* = 0.05, 0.1, 0.15, 0.2): (a) O 1s, (b) Co 2p.

XPS is performed to determine the surface elemental valence states of NSC_*x*_C series materials. As shown in Fig. 6(a), deconvolution is performed on the O 1s peak of the NSC_*x*_C material. The O 1s shows a high peak and a low peak that are fitted to another four peaks, namely, moisture oxygen (O_moisture_), adsorbed oxygen (O_adsorbed_), vacancy oxygen (O_vacancy_), and lattice oxygen (O_lattice_). The corresponding binding energy of each peak is shown in Table 3.^14^ With the increasing temperature, the oxygen in O_adsorbed_ and O_vacancy_ is easily removed from the lattice and thus creates oxygen vacancies. The ratio of (O_adsorbed_ + O_vacancy_)/O_lattice_ is therefore calculated and shown in Table S2.† The ratio of (O_adsorbed_ + O_vacancy_)/O_lattice_ increases at *x* ≤ 0.1, indicating that Ca introduction can increase the generation of oxygen vacancies. When *x* ≥ 0.1, the reduced ratio is attributed to the additional rock salt-like layer of the newly generated NdCaCoO_4+*δ*_ compared with the original phase; this layer increased the overall lattice oxygen content.^32,33^ According to current oxygen ion conduction theory, the increase in oxygen vacancies can improve the oxygen ion migration ability during ORR, and the higher adsorbed oxygen content can promote the migration of oxygen to triple-phase boundary (TPB) and the diffusion rate at the electrolyte–cathode interface. The XPS plots of Co 2p for NSC_*x*_C are demonstrated in Fig. 6(b). The curve shows two peaks with high (Co 2p_1/2_) and low (Co 2p_3/2_) binding energies. The Co 2p_3/2_ peaks are classified as Co^3+^ and Co^4+^ according to their binding energies of 779.9 ± 0.2 and 780.8 ± 0.2 eV, respectively, and the Co 2p_1/2_ peaks are classified as Co^3+^ and Co^4+^ according to their binding energies of 794.9 ± 0.2 and 796.4 ± 0.2 eV, respectively.^14,31,34,35^ The binding energy positions and Co^3+^/Co^4+^ contents are shown in Table 4. With the increase in Ca content, Co^3+^ increases and Co^4+^ decreases, thus further improving the electrochemical performance of the materials.

**Table 3:** The binding energy of O_moisture_, O_adsorbed_, O_vacancy_, O_lattice_ and the ratio of (O_adsorbed_ + O_vacancy_)/O_latticee_

Sample	O_moisture_ (eV)	O_adsorbed_ (eV)	O_vacancy_ (eV)	O_lattice_ (eV)	(O_adsorbed_ + O_vacancy_)/O_lattice_
NSC	532.85	531.19	529.25	528.63	3.14
NSC_0.05_C	532.90	531.08	529.31	528.38	3.29
NSC_0.1_C	532.72	531.20	529.34	528.54	3.35
NSC_0.15_C	532.92	531.20	529.12	528.50	3.32
NSC_0.2_C	532.94	531.25	529.38	528.58	3.30

**Table 4:** The Co2p binding energy and the content of Co^4+^ and Co^3+^ calculated from the corresponding XPS peaks

Sample	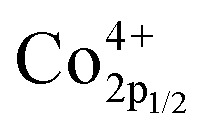 (eV)	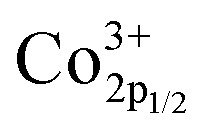 (eV)	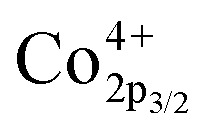 (eV)	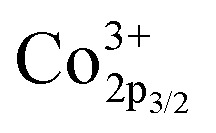 (eV)	Co^4+^ (%)	Co^3+^ (%)
NSC	796.53	795.05	780.87	779.95	65.87	34.13
NSC_0.05_C	796.38	794.82	780.64	779.75	57.88	42.12
NSC_0.1_C	796.3	794.89	780.66	779.84	50.28	49.72
NSC_0.15_C	796.30	794.89	780.65	779.89	48.50	51.50
NSC_0.2_C	796.64	795.02	780.78	779.95	47.76	52.24

**Fig. 7:**
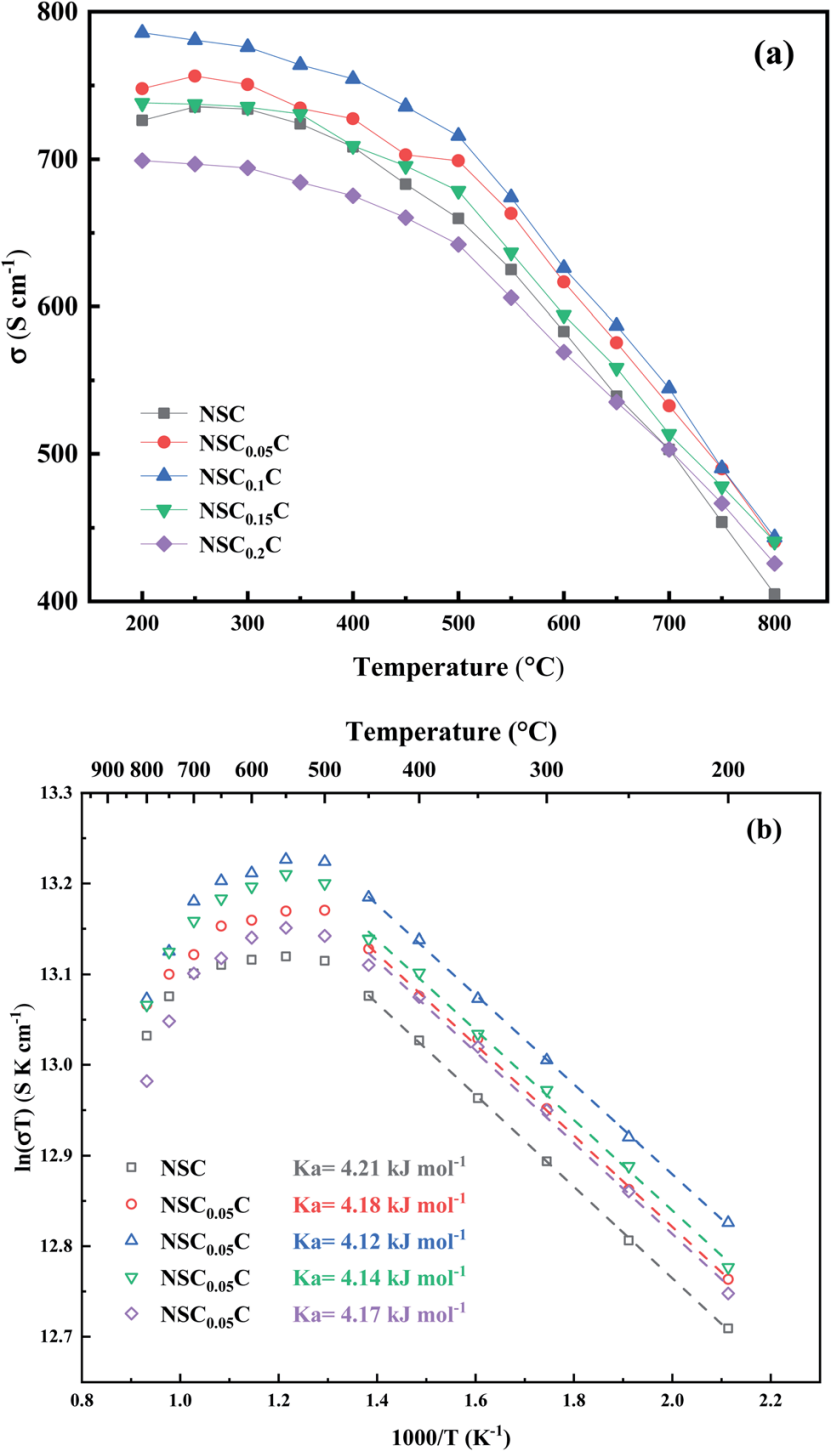
(a) Electrical conductivity; (b) Arrhenius plots of NSC_*x*_C(*x* = 0.05, 0.1, 0.15, 0.2).

The temperature dependence of the conductivity curve of NSC_*x*_C in the range of 200–800 °C is shown in Fig. 7(a). In perovskite structure, the electronic conductivity is higher than the ionic conductivity and therefore is the dominant force.^5,36^ The joint action of the two compensation modes leads to the phenomenon that the conductivity decreases with the increase of temperature. With the increase of temperature, ion conduction begins to play a role, and the effect of electron conduction weakens. At the same temperature, the conductivity first increases with the increasing doping concentration of Ca^2+^ and reaches the peak at 0.1, indicating a typical property of semiconductor. But when the Ca^2+^ doping concentration further increases, the conductivity decreases, indicating a metallic conductive behavior.^28^ In perovskite oxides, electron conduction relies mainly on the B–O–B conduction mechanism, which is determined by the overlap of the 2p orbital of oxygen with the 3d orbital of transition metal.^37,38^ Ca^2+^ doping creates additional holes, which strengthens the electron conduction mechanism:1
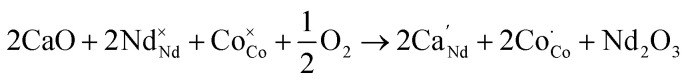


But the doping of Ca^2+^ also leads to the generation of extra oxygen vacancies, which hinder the transition of electrons, and decreases the conductivity:2
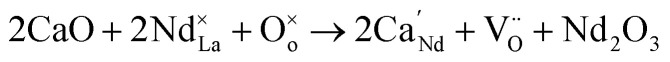


The combined action of two mechanisms increases the electrical conductivity when the Ca^2+^ doping concentration is appropriate for the NSC_*x*_C. Goldschmidt tolerance factor is calculated by eqn (3):3
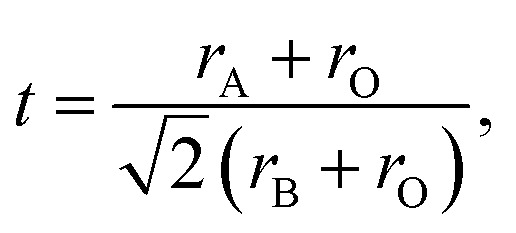
where *r*_A_, *r*_B_, and *r*_O_ are the radii of A^3+^, B^3+^, and O^2−^ ions, respectively. The tolerance factors of NSC, NSC_0.05_C, NSC_0.1_C, NSC_0.15_C, and NSC_0.2_C are 0.9513, 0.9527, 0.9541, 0.9555 and 0.9569, respectively. The *t*-value increases from 0.9513 (NSC) to 0.9569 (NSC_0.2_C) with the substitution of Ca for Nd. This phenomenon occurs because the substitution of Ca at 0.1 increases the bond angle of O–Co–O over 180° and further increases the degree of O(2p) stacking with Co(3d), thereby enhancing the Co–O covalency, proton conduction, and conductivity.^14,27^ When the temperature increases, the Ca-induced enhancement on conductivity decreases compared with that at low temperature state. This finding can be attributed mainly to Co^3+^ stabilization in the intermediate spin state caused by Ca^2+^ addition in the hole-conducting mechanism; the result is the formation of oxygen vacancies.^39^ When *x* > 0.1, the rock salt-like layer in the latest phase restricts the proton conduction path; hence, the conductivity shows a decreasing trend.^40,41^ The Arrhenius plots of the NSC_*x*_C cathode are shown in Fig. 7(b). The small polariton activation energy *E*_a_ is calculated by eqn (4) and placed in the plot:4
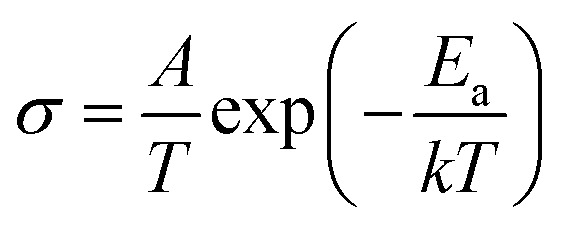
where *A* is the pre-exponential factor, *T* is the absolute temperature, *k* is the Boltzmann constant and *E*_a_ is the activation energy. The results show that the small polaron activation energy *E*_a_ first decreases then increases when the Ca content increases. The substitution of Ca^2+^ for Nd^3+^ enhances the proton transport and constrains the oxygen loss; as a result, the activation energy decreases slightly under the combined effect of electron compensation and ion compensation.^27^ By contrast, when *x* > 0.1, contribution to the electrode reaction, indicating that the lower second phase may not form a connected heterogeneous interface. Therefore, its reduced value can be attributed to the decrease in oxygen vacancy caused by the increase in Ca^2+^; as a result, the activation energy and energy barrier increase.^20^

**Fig. 8:**
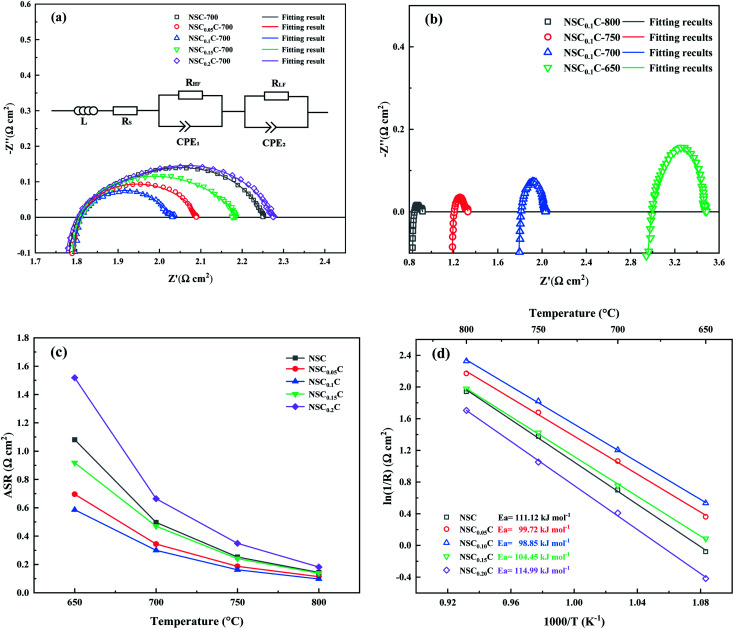
(a) Impedance spectra measured at 700 °C in the air on symmetric cells with NSC_*x*_C(*x* = 0.05, 0.1, 0.15, 0.2); (b) impedance spectra measured in the range of 650–800 °C; (c) ASR values of NSC_*x*_C (*x* = 0.05, 0.1, 0.15, 0.2) at 650–800 °C; (d) Arrhenius plots of ln(1/*R*) *vs.* 1000/*T* for NSC_*x*_C (*x* = 0.05, 0.1, 0.15, 0.2) in air.

The effect of Ca doping on the performance of NSC_*x*_C cathode is investigated by EIS. Fig. 8(a) shows the Nyquist diagram for NSC_*x*_C materials with different Ca contents at 700 °C. All the curves show semicircular arcs of different radii and reflect the specific electrochemical processes at a specific temperature. In general, the two arcs correspond to high and low frequency, respectively. The polarization resistance of the arc in the high frequency section is influenced by the charge transfer of oxide ions at the interface of electrode and electrolyte. Meanwhile, the arc in the low frequency region corresponds to oxygen adsorption and dissociation. The Nyquist curves for NSC_0.1_C at different temperatures are also displayed in Fig. 8(b). The increasing temperature advances the electrocatalytic performance of the cathode, resulting in a smaller arc. The Nyquist curves at other temperatures and their fits are shown in Fig. S2(a–c),† and the polarization resistance (*R*_p_) is calculated from these fits and normalized to obtain the area specific resistance (ASR) with the value placed in Fig. 8(c). With the increase in Ca doping, the ASR first decreases and then increases. ASR is the lowest at all temperature points when *x* = 0.1 but starts to increase when *x* > 0.1. The Arrhenius plots of NSC_*x*_C at 800–650 °C are drawn using the ASR values obtained in the EIS tests as shown in Fig. 8(d). The activation energy *E*_a_ for each material is further calculated by the Arrhenius equation as listed in Fig. 8(d). The symmetric cell exhibits the lowest activation energy of 98.85 kJ mol^−1^ when *x* = 0.1. The reduced activation energy at *x* ≤ 0.1 can be attributed to the following: the replacement of Nd^3+^ by Ca^2+^ creates additional oxygen vacancies, increases the oxygen transport rate, expands the adsorption/dissociation pathway, and improves its oxygen adsorption/dissociation and oxygen ion surface diffusion as a cathode material.^14,42^ By contrast, when *x* > 0.1, its ASR and *E*_a_ increase, indicating that the spin state of Co^3+^ is induced by the increasing Ca^2+^ content at high temperatures, thus limiting further the generation of oxygen vacancies.^21,31,39^

**Fig. 9:**
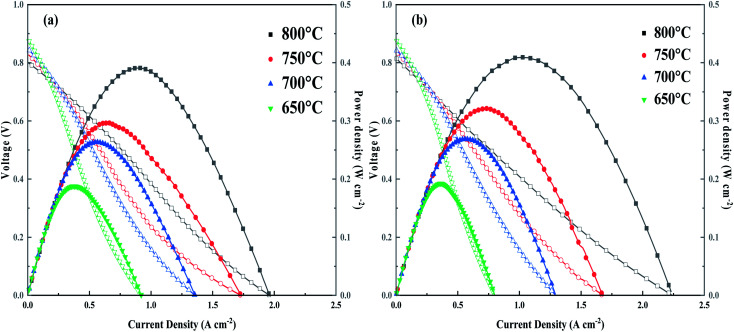
I–V–P performance of the single cell with (a) NSC; (b) NSC_0.1_C.

The I–V–P curves with corresponding power density curves for NSC (a) and NSC_0.1_C (b) at 800–600 °C are depicted in Fig. 9. The OCV is approximately 0.8 V at 800 °C and increases to approximately 0.9 V at 600 °C. The maximum power densities of NSC_0.1_C at 600 °C, 650 °C, 700 °C, 750 °C, and 800 °C are 409.31, 321.40, 268.69, 192.44, and 158.10 mW cm^−2^, respectively. The output power density of NSC at 800 °C is 391.03 mW cm^−2^, which is lower than that of NSC_0.1_C at all other temperatures (Table S3†). Hence, the power density at *x* = 0.1 is slightly higher than that of the sample without Ca doping. Therefore, NSC_0.1_C is a promising cathode material for IT-SOFC.

## 4. Conclusions

The effects of Ca doping on it's the structural and electrochemical properties of Nd_0.8_Sr_0.2_CoO_3−*δ*_ were investigated, and its performance as an IT-SOFC cathode material was evaluated. When the Ca content increases, the unit cell size of Nd_0.8−*x*_Sr_0.2_Ca_*x*_CoO_3−*δ*_ increases and CTE decreases. Nd_0.7_Sr_0.2_Ca_0.1_CoO_3−*δ*_ shows good electrochemical performance, and its conductivity decreases with the increase of temperature, and it is 443 S cm^−1^ at 800 °C. However, low area-specific resistance values are obtained due to the dominance of ionic compensation. At *x* = 0.1, its area specific resistance is 0.0976 Ω cm^2^ at 800 °C, implying good oxygen diffusion. The maximum power density of Nd_0.7_Sr_0.2_Ca_0.1_CoO_3−*δ*_ is 409.31 mW cm^−2^ at 800 °C, which is slightly higher than that of Nd_0.8_Sr_0.2_CoO_3−*δ*_ at 391.03 mW cm^−2^. This finding indicates that the CTE of Nd_0.7_Sr_0.2_Ca_0.1_CoO_3−*δ*_ can be reduced while maintaining a power density similar to that of the original structure. Therefore, Nd_0.7_Sr_0.2_Ca_0.1_CoO_3−*δ*_ has good electrochemical properties and can be used as a candidate material for IT-SOFC cathodes.

## Conflicts of interest

There are no conflicts to declare.

## Supplementary Material

RA-012-D2RA02546A-s001
