# Supplementary material for: Evaluation of Nd0.8−xSr0.2CaxCoO3−δ(x = 0, 0.05, 0.1, 0.15, 0.2) as a cathode material for intermediate-temperature solid oxide fuel cells
Source: RSC Adv. 2022 Jun 13;12(27):17379–89. doi: 10.1039/d2ra02546a (PMC9190786; doi:10.1039/d2ra02546a)
Supplement: RA-012-D2RA02546A-s001 [file RA-012-D2RA02546A-s001.pdf]

Electronic Supplementary Material (ESI) for RSC Advances.
This journal is © The Royal Society of Chemistry 2022

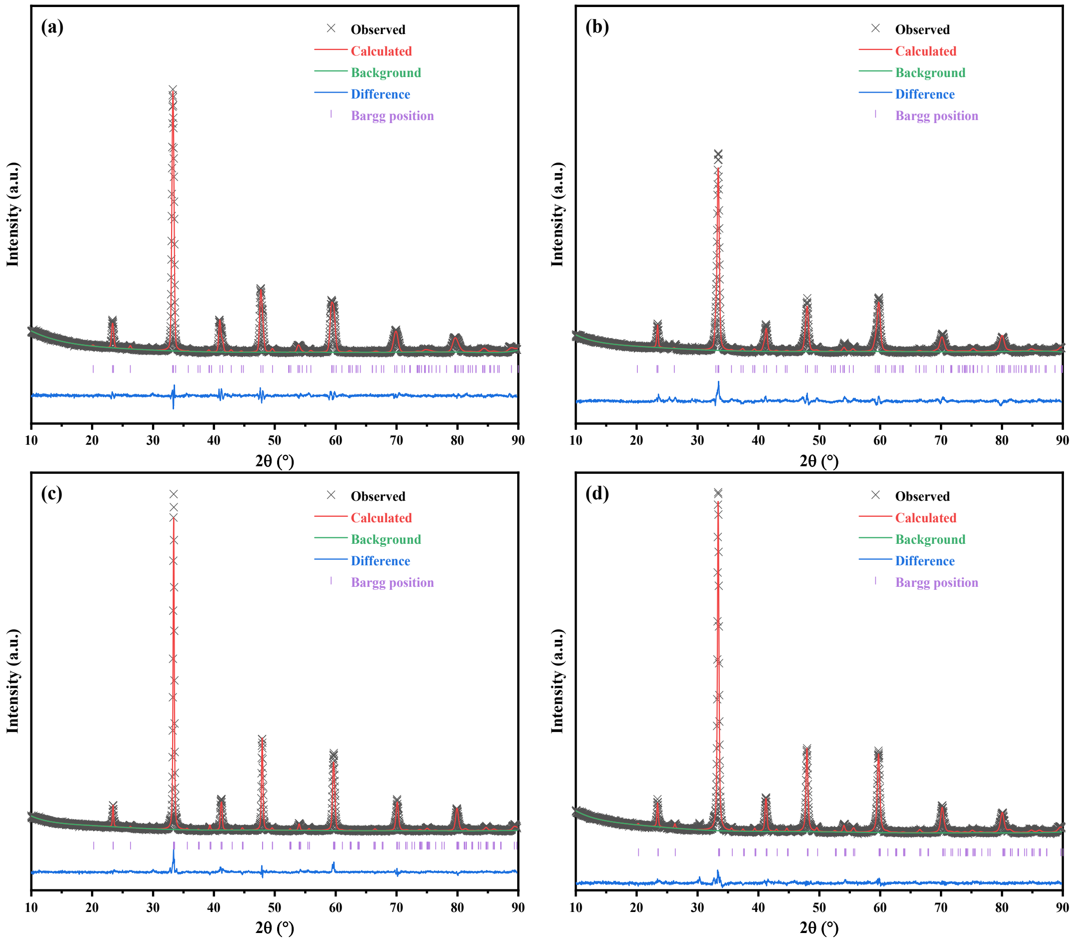

Fig.S1 Rietveld refined XRD of NSC_xC: (a) NSC; (b) $NSC_{0.05}C$; (c) $NSC_{0.15}C$; (d) $NSC_{0.2}C$

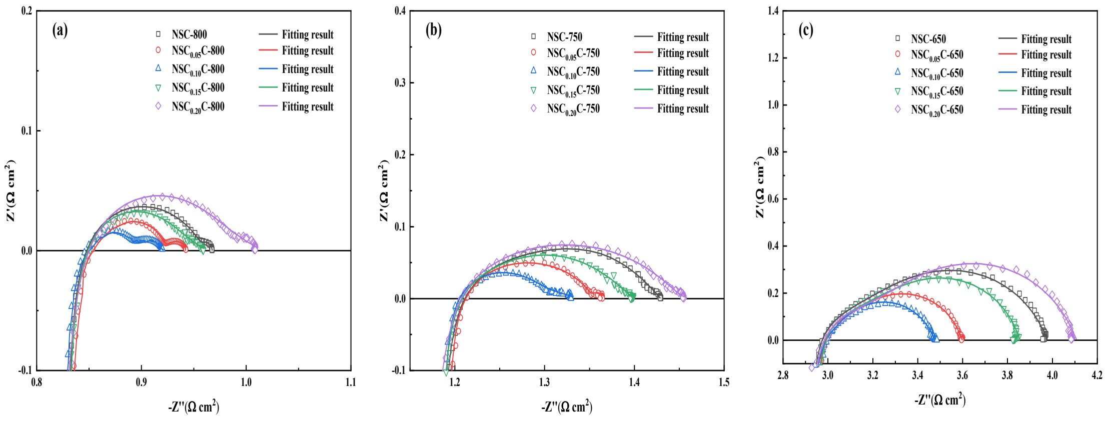

Fig.S2 Nyquist diagram of NSC$_x$C (a)800 ℃; (b) 750 ℃; (c) 700 ℃

Table S1 Thermal expansion coefficient of NSC_xC at different temperatures

Sample	CTE($\times 10^{-6}$ ℃)				
	35~800 ℃	35~200 ℃	200~400 ℃	400~600 ℃	600~800 ℃
NSC	24.2049	18.1394	25.9635	25.8948	25.7603
$NSC_{0.05}C$	22.5204	18.8576	22.4666	23.7632	24.3532
$NSC_{0.1}C$	21.7031	17.1650	21.6936	23.0238	24.1359
$NSC_{0.15}C$	21.2729	16.7099	20.4958	22.5887	24.0989
$NSC_{0.2}C$	19.7623	16.8623	17.8104	19.9381	23.9310

Table S2 The binding energy of $O_{moisture}$, $O_{adsorbed}$, $O_{vacancy}$, $O_{lattice}$ and the ratio of ($O_{adsorbed}$+$O_{vacancy}$)/$O_{latticee}$.

Sample	$O_{moisture}$(eV)	$O_{adsorbed}$(eV)	$O_{vacancy}$(eV)	$O_{lattice}$(eV)	($O_{adsorbed}$+$O_{vacancy}$)/$O_{lattice}$
NSC	532.85	531.19	529.25	528.63	3.14
$NSC_{0.05}C$	532.90	531.08	529.31	528.38	3.29
$NSC_{0.1}C$	532.72	531.20	529.34	528.54	3.35
$NSC_{0.15}C$	532.92	531.20	529.12	528.50	3.32
$NSC_{0.2}C$	532.94	531.25	529.38	528.58	3.30

Table S3 Power density of NSC and $NSC_{0.1}C$ in range of 800-650 ℃

Sample	800℃($mW.cm^{-2}$)	750℃($mW.cm^{-2}$)	700℃($mW.cm^{-2}$)	650℃($mW.cm^{-2}$)
NSC	391.03	296.30	263.25	187.85
$NSC_{0.1}C$	409.31	321.40	268.69	192.44
